# Diagnostic and Therapeutic Approach to External Frontal Lesions: A Report of Two Cases

**DOI:** 10.7759/cureus.104060

**Published:** 2026-02-22

**Authors:** Luis F Ochoa, Vanessa Galvan, Carlos A Garay, Francisco Diaz, Aurora Rico, Alejandro Ovando, Natalia Bezies, Alisson C Aguirre

**Affiliations:** 1 Surgery, Hospital General Instituto de Seguridad y Servicios Sociales de los Trabajadores del Estado (ISSSTE) Presidente General Lázaro Cárdenas del Río, Chihuahua, MEX; 2 Surgery, Instituto Mexicano del Seguro Social (IMSS) Hospital General Regional #1, Ciudad Obregon, MEX; 3 General Surgery, Hospital General de Tijuana, Tijuana, MEX; 4 School of Medicine, Universidad Autonoma de Baja California, Mexicali, MEX; 5 School of Medicine, Universidad de las Americas, Quito, ECU

**Keywords:** ambulatory surgery, frontal lipoma, frontal sinus osteoma, plastic and reconstructive surgery, surgical extraction

## Abstract

Benign frontal masses represent a diagnostic and therapeutic challenge due to their aesthetic impact and the wide range of possible differential diagnoses. We present a comparative case series of two patients with benign frontal masses: a frontal lipoma and a frontal osteoma. Both cases highlight the importance of careful clinical evaluation, appropriate use of imaging studies, and tailored surgical planning to achieve optimal functional and aesthetic outcomes. Frontal osteomas are benign, slow-growing bone tumors arising from the cranial vault and represent the most common benign tumors of the skull. Although often asymptomatic, their frontal location frequently leads to aesthetic deformity and patient concern, prompting surgical evaluation. Lipomas are the most common benign soft-tissue tumors, but their presentation in the frontal region is relatively rare. Because of their location, they are often confused with other lesions, such as osteomas or epidermoid cysts.

## Introduction

Osteomas are benign osteogenic tumors characterized by the proliferation of mature compact or cancellous bone. They commonly involve the craniofacial skeleton, particularly the frontal bone, and represent the most frequent benign tumors of the cranial vault [1,2]. Frontal osteomas typically arise from the outer table of the frontal bone and exhibit slow, progressive growth over several years. Although many osteomas remain asymptomatic, lesions located in the frontal region frequently result in visible contour deformities, leading patients to seek medical attention for aesthetic concerns or localized discomfort [3]. Clinically, these tumors present as hard, immobile masses fixed to the underlying bone, which aids in distinguishing them from soft-tissue lesions; however, imaging is essential for definitive diagnosis and surgical planning [4].

Lipomas are benign tumors composed of mature adipocytes that typically develop in subcutaneous tissue, although they may also be located within muscular planes or deeper anatomical layers [5]. While lipomas are common in the trunk and extremities, they account for only 10-15% of cases in the head and neck region, with frontal localization particularly rare [6]. Clinically, frontal lipomas usually present as soft, mobile, asymptomatic, and slowly growing lesions [7].

Given the overlapping clinical presentation of slow-growing frontal masses, accurate preoperative differentiation between osteomas and lipomas is essential for appropriate surgical planning and patient counseling. Imaging studies play a crucial role in this distinction: ultrasonography typically demonstrates a homogeneous, hyperechoic mass in lipomas; computed tomography is particularly useful for identifying bone involvement characteristic of osteomas; and magnetic resonance imaging can confirm adipose content by demonstrating a hyperintense signal on T1-weighted sequences [8].

The treatment of choice for both entities is complete surgical excision, either for aesthetic concerns, symptomatic relief, or definitive histopathological confirmation [9]. Recent reviews report recurrence rates of less than 5% following adequate excision of lipomas, confirming the effectiveness of surgical management [10]. Therefore, the primary objective of this study is to highlight the clinical and radiological features that allow accurate differentiation between frontal masses, thereby facilitating appropriate diagnosis, surgical planning, and optimal patient outcomes.

## Case presentation

Case 1

**Figure 1:**
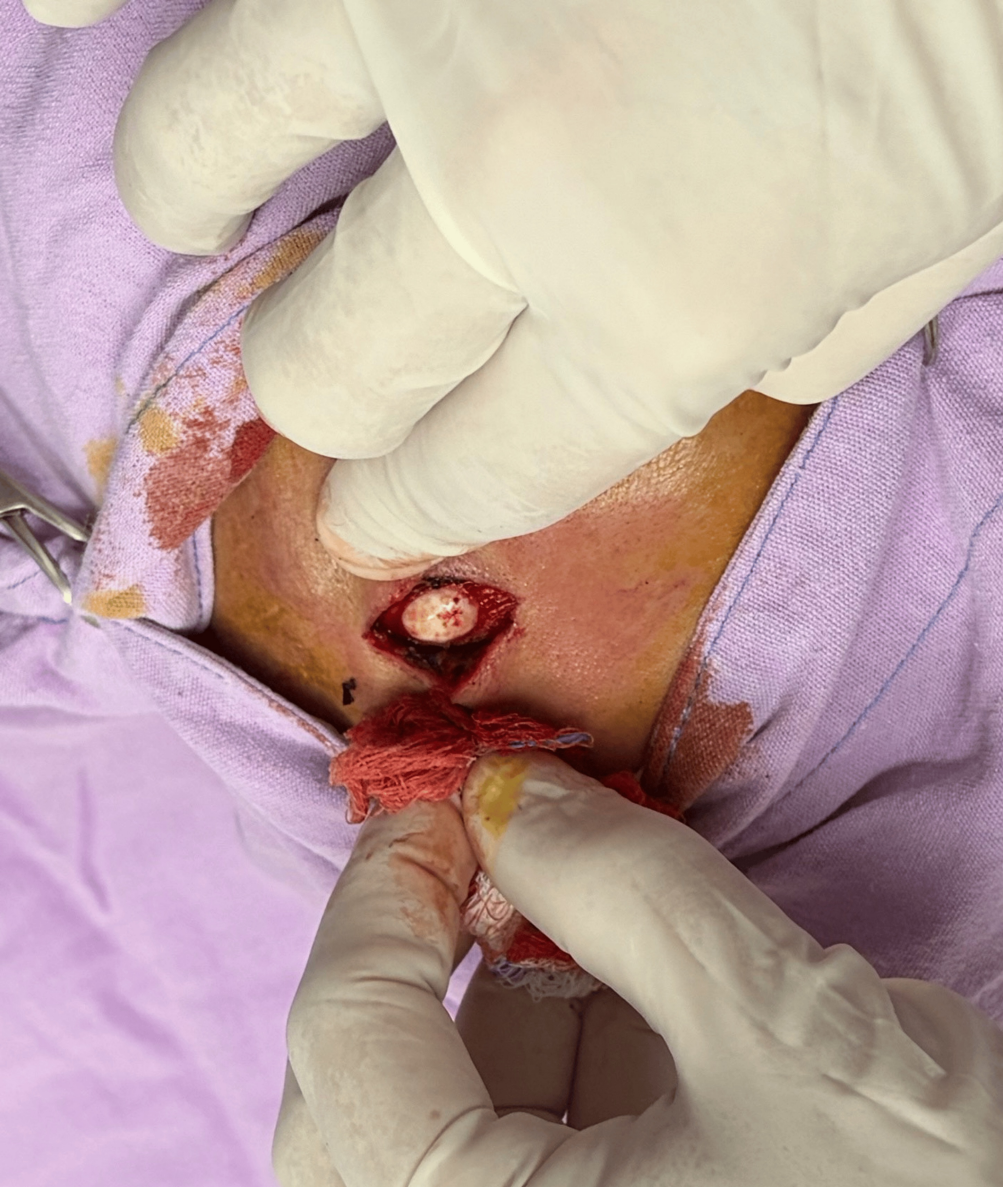
Frontal osteoma A whitish lesion was observed; on palpation, it was found indurated and firm, originating from the frontal bone.

A 44-year-old male patient presented with a mass located in the midline frontal region, with a reported evolution time of approximately three years. The lesion had progressively increased in size and was associated with occasional mild discomfort. The primary concern was aesthetic, as the deformity was clearly visible and affected facial symmetry. Physical examination revealed a well-defined, hard, immobile mass fixed to the frontal bone, with normal overlying skin and no signs of inflammation. Neurological examination was normal. The diagnosis was established solely on clinical grounds, without the need for imaging studies. Surgical excision was indicated due to aesthetic deformity. The procedure was performed under local anesthesia using a direct incision placed along a natural frontal skin crease (Figure 1); it was excised using a gouge, followed by careful smoothing of the frontal bone contour. The subcutaneous tissue was closed using skin absorbable suture and the skin with non-absorbable suture. The postoperative course was uneventful, with satisfactory aesthetic results. Histopathological examination demonstrated bone tissue associated with a frontal osteoma.

Case 2

A 38-year-old female patient, with no significant past medical history, presented with a mass in the right frontal region of two years' duration. The lesion had progressively increased in size and volume and was later accompanied by associated symptoms, predominantly frontal pain radiating to the right periorbital area. The patient also reported that the mass's aesthetic impact interfered with her daily activities. The diagnosis was established solely on clinical grounds, without the need for imaging studies. On physical examination, an evident mass was observed in the frontal region. Palpation revealed a soft, non-indurated, and mobile lesion, highly suggestive of a lipoma. The lesion was previously marked

**Figure 2:**
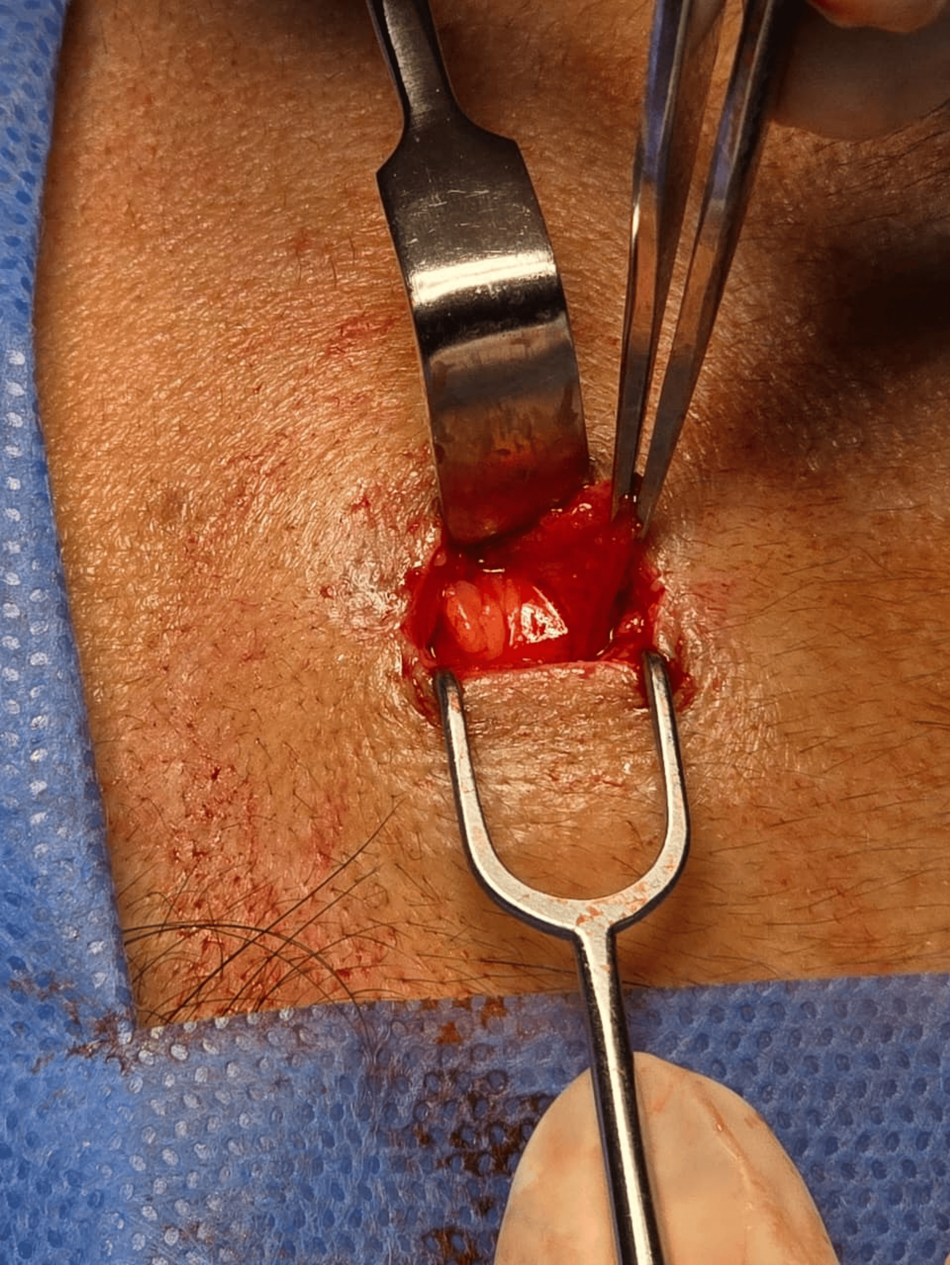
Frontal lipoma A yellowish lesion was observed; on palpation, it was found to be soft and mobile, arising from the subcutaneous tissue.

**Figure 3:**
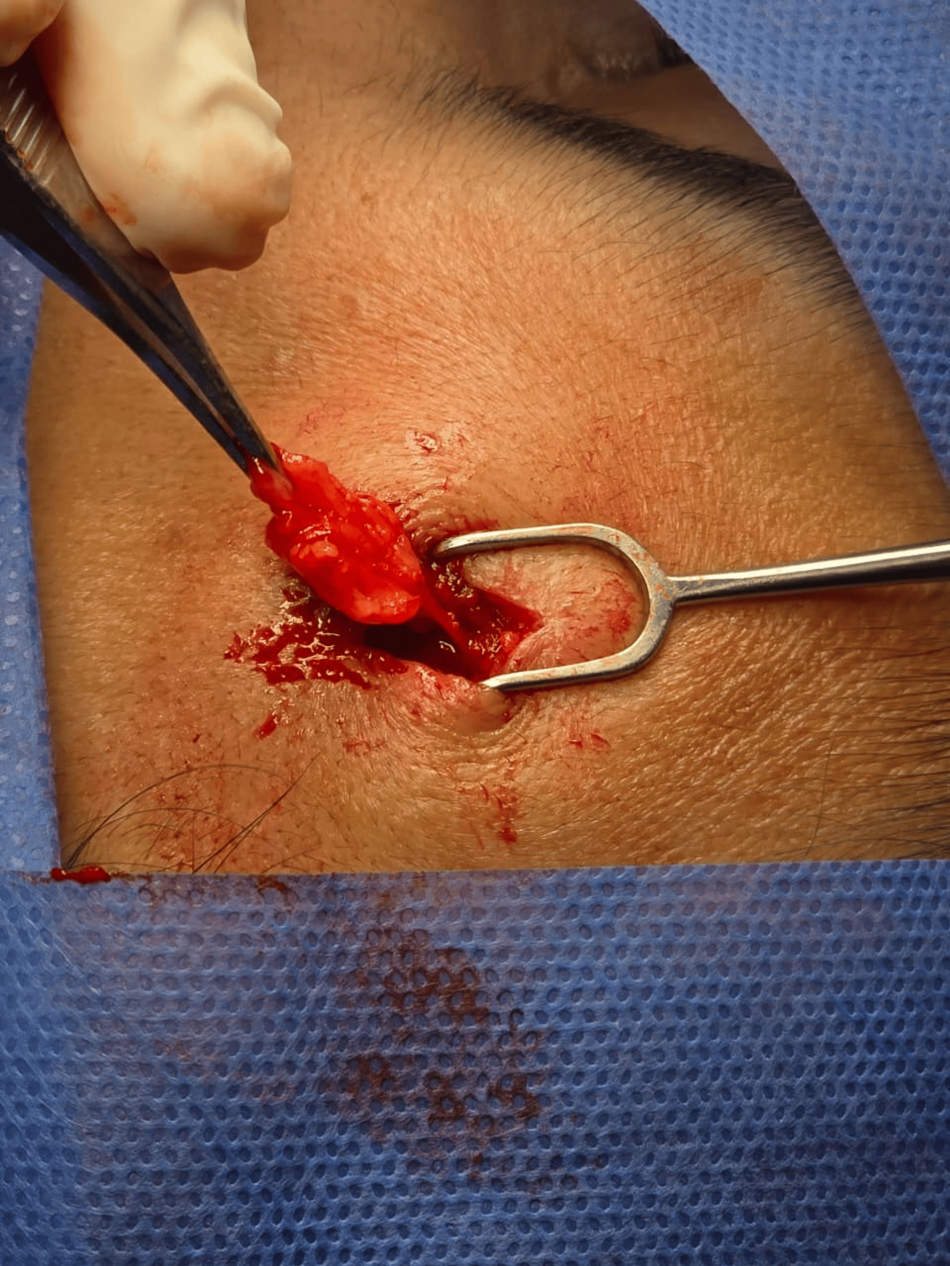
Frontal lipoma Complete resection of the adipose tissue can be observed, confirming the macroscopic diagnosis of a frontal lipoma.

Surgical excision was performed through a direct incision along a natural frontal skin crease. Intraoperatively, the lesion was identified with immediate recognition of adipose tissue content (Figure 2). Complete excision of the encapsulated lipoma was achieved (Figure 3) without injury to the underlying frontalis muscle or neurovascular structures. Histopathological findings demonstrated the presence of fatty tissue consistent with a frontal lipoma.

## Discussion

Frontal osteomas are slow-growing benign tumors arising from membranous bone and most commonly involving the craniofacial skeleton, particularly the frontal bone [1,2]. Several etiological theories have been proposed, including embryologic developmental anomalies, trauma, and chronic inflammatory processes; however, no definitive cause has been established [3]. Clinically, frontal osteomas present as painless, firm, immobile masses fixed to the underlying bone, distinguishing them from lipomas, which are typically soft, mobile, and compressible [4,5]. Nevertheless, clinical examination alone may be insufficient, and imaging plays a critical role in diagnosis and surgical planning. Computed tomography is considered the gold standard for diagnosing frontal osteomas, as it provides excellent visualization of bone density and architecture, confirms the osseous origin of the lesion, and excludes sinus or intracranial involvement [8]. Magnetic resonance imaging is generally unnecessary unless atypical features or soft tissue extension are suspected.

Surgical excision is indicated in cases of symptomatic lesions, progressive growth, or significant aesthetic deformity. Direct surgical approaches allow precise removal and restoration of forehead contour, it also provides direct visualization, complete lesion exposure, and facilitates management of large or complex tumors; however, it is associated with larger incisions, more visible scarring, increased soft tissue dissection, and potentially longer recovery times [7]. Alternative approaches, such as endoscopic or coronal techniques, may be considered for larger or less accessible lesions; it has superior aesthetic outcomes, smaller incisions, reduced tissue trauma, and faster recovery, but they may have limited exposure, a steeper learning curve, and technical constraints when managing large or deeply attached lesions. The choice of approach should be individualized based on tumor size, location, surgeon experience, and patient aesthetic expectations. Complete excision is associated with excellent outcomes and low recurrence rates, and malignant transformation of osteomas has not been reported, reinforcing their benign nature and favorable prognosis [1,11,12].

Frontal lipomas, although relatively rare, are an important entity in the differential diagnosis of soft tissue masses of the forehead. Their slow growth, soft consistency, and mobility over deep planes are characteristic clinical features; however, their location may lead to misdiagnosis as osteomas, epidermoid cysts, dermoid cysts, or other benign and malignant lesions [4,9]. Imaging modalities, although not always required for superficial lesions, can provide valuable information in atypical or larger masses. Ultrasonography is a simple, non-invasive tool that typically demonstrates a homogeneous, hyperechoic lesion, whereas computed tomography and magnetic resonance imaging are indicated when deeper involvement or bone erosion is suspected and when a complete physical examination has been performed but diagnostic doubts remain [5,6,11,12]. In the present case, the lesion was clinically typical of a lipoma, and imaging was deemed unnecessary, illustrating that careful physical examination remains crucial in clinical decision-making.

Surgical excision remains the treatment of choice, providing definitive diagnosis and resolution of aesthetic or functional concerns. Complete removal of the encapsulated lesion is associated with excellent outcomes and low recurrence rates, generally reported to be less than 5% [4,6,9]. Additionally, the frontal location poses specific considerations: careful planning of the incision along natural skin creases minimizes visible scarring, and meticulous dissection helps preserve underlying structures, including the frontalis muscle and neurovascular bundles [13].

From a pathophysiological perspective, lipomas are composed of mature adipocytes, often encapsulated, and their exact etiology is not fully understood. Genetic predisposition, minor trauma, and metabolic factors have been proposed as contributing mechanisms [14,15]. Although malignant transformation is exceedingly rare, clinicians must remain vigilant, particularly in lesions with rapid growth, pain, or firm consistency, which may warrant histopathological evaluation to exclude liposarcoma [16]. Regarding other possible etiologies, we need to know the differential diagnoses (Table 1).

**Table 1:** Differential diagnosis of external frontal masses

Characteristic	Lipoma	Osteoma	Epidermoid cyst
Frequency	Relatively uncommon in the forehead; more frequent in trunk and extremities [6,9]	Most common benign skull lesion; frequent in the forehead [1,2]	Relatively common in face and scalp [17,18]
Tissue of origin	Adipose tissue composed of mature adipocytes [5,14]	Bone tissue, usually arising from the outer table of the frontal bone [1,2,12]	Squamous epithelium producing keratin [17,18]
Consistency	Soft, compressible [5,6]	Very hard, stony [1,3]	Firm, elastic; may fluctuate if keratin content is present [17]
Mobility	Mobile over deep planes (unless subgaleal or intramuscular) [5,9]	Fixed to bone, immobile [1,4]	Generally mobile but adherent to skin [17,18]
Growth	Slow, painless [6,10]	Very slow, usually incidental [2,3]	Slow; may become inflamed or infected [17,18]
Symptoms	Generally asymptomatic; cosmetic concern [6,9]	Asymptomatic; mainly cosmetic concern [1,2]	May be painful or erythematous if infected [17,18]
Imaging	Ultrasound: homogeneous hypo/hyperechoic mass; MRI: T1 hyperintense [8,9]	CT: well-defined bone density lesion [6,12]	Ultrasound: well-defined lesion with keratin content [17,18]
Treatment	Surgical excision if aesthetic concern or symptomatic; low recurrence [6,9,10]	Surgical removal (osteotomy/curettage) if symptomatic or deforming [7,11]	Complete excision with capsule to prevent recurrence [17,18]

Both cases highlight several important points: the value of detailed clinical assessment, the appropriateness of conservative imaging strategies when the diagnosis is evident, and the importance of aesthetic and functional considerations in surgical planning.

Study limitations

This study has several limitations inherent to its design as a report of two cases. The findings cannot be generalized due to the absence of a control group and the limited sample size, which precludes statistical analysis or causal inference. Additionally, follow-up was limited, restricting the assessment of long-term outcomes. Despite these constraints, the report provides clinically relevant insights into the diagnostic challenges and surgical management of frontal masses, contributing to the existing literature and supporting the need for further comparative studies with larger cohorts; clinicians should know the differential diagnosis of forehead masses and consider patient-centered surgical planning to optimize outcomes.

## Conclusions

Frontal osteoma is a benign but clinically relevant condition that should be considered in the differential diagnosis of forehead masses. Accurate diagnosis relies on careful clinical assessment and computed tomography, which remains the imaging modality of choice. When aesthetic deformity or symptoms are present, surgical excision, performed with a well-planned approach, offers excellent cosmetic and functional outcomes with minimal morbidity. These cases highlight the importance of individualized surgical planning and reinforce the view of frontal osteoma as a distinct entity requiring a structured diagnostic and therapeutic approach. Frontal lipomas are uncommon but clinically significant benign soft tissue tumors. Accurate diagnosis relies primarily on careful physical examination, with imaging reserved for atypical presentations or suspicion of deeper involvement. Surgical excision is the definitive treatment, offering both aesthetic and functional benefits with minimal recurrence risk.
